# An Analysis of Rabies Incidence and Its Geographic Spread in the Buffer Area Among Orally Vaccinated Wildlife in Ukraine From 2012 to 2016

**DOI:** 10.3389/fvets.2019.00290

**Published:** 2019-09-10

**Authors:** Ivan Polupan, Maksym Bezymennyi, Yurii Gibaliuk, Zhanna Drozhzhe, Oleksii Rudoi, Vitalii Ukhovskyi, Vitalii Nedosekov, Marco De Nardi

**Affiliations:** ^1^Department of Research Virology, State Scientific Research Institute of Laboratory Diagnostics and Veterinary and Sanitary Expertise, Kyiv, Ukraine; ^2^GIS Department, Institute of Veterinary Medicine of the National Academy of Agrarian Sciences of Ukraine, Kyiv, Ukraine; ^3^State Service of Ukraine for Food Safety and Consumer Protection, Kyiv, Ukraine; ^4^Department of Epizootology and Veterinary Business Organization, National University of Life and Environmental Sciences of Ukraine, Kyiv, Ukraine; ^5^SAFOSO AG, Liebefeld, Switzerland

**Keywords:** Ukraine, rabies, surveillance, rabies case, fox, oral rabies vaccination

## Abstract

The statistics of rabies cases in Volyn, Lviv, and Zakarpattia oblasts of Ukraine from 2012 to 2016 were analyzed to establish spatial–temporal distribution of rabies endemic outbreaks and to identify causes of widespread infections among wild and domestic animals. The occurrence of rabies outbreaks in wild and domestic animals in Ukraine was also assessed to determine the effectiveness of oral rabies vaccination (ORV) efforts. According to our analysis, parenteral vaccination of domestic animals and ORV campaigns in foxes have proved unsuccessful in providing a sustainable, long-term reduction in endemic rabies outbreaks. ORV campaigns in foxes were deemed ineffective based on our studies of the endemic rabies outbreaks in Volyn, Lviv, and Zakarpattia oblasts in 2012–2016. The current rabies prevention system (parenteral vaccination) failed to offer protection to domestic animals based on our review of the occurrence of endemic rabies outbreaks in dogs and cats. ORV campaign shortcomings and their causes must be identified in order to provide maximum rabies vaccine coverage for dogs and cats. Altogether, the results presented here provide information that can assist public health agencies to devise more effective disease control plans to curtail the spread of rabies in domestic animals and wildlife in Ukraine.

## Introduction

In the early twenty first century, rabies still represents a significant veterinary and public health concern in Ukraine (UA). The disease is detected throughout the UA territory, and among all European countries, UA presents with the highest incidence of rabies cases (1).

Few cases of rabies were detected in domestic animals until the mid-1960s in UA. In 1965, for example, 140 cases of rabies were recorded among animals (2). In the 1970s, new high endemic rabies incidence was shown to coincide with the introduction of rabies carried by red foxes (*Vulpes vulpes*) (3, 4).

From 1970 to 2000, rabies incidence rates fluctuate in UA with an annual maximum recording of 1,500 cases in animals. Outbreaks of rabies in animals were reported in 1979, 1984, and 1990 (5). In 2003, 2005, 2006, 2007, and 2008, over 2,000 rabies cases per year were recorded. An additional 37% of rabies cases were found for foxes. Multiple factors cause rabies in domestic and farm animals. The most important factors appear to be the ecology of foxes, their synantropy, high densities (5–6 foxes/100 km^2^), the presence and density of roaming dogs, and the contact of foxes with dogs and cats (6). Another reason for the rabies endemic is low percentage of immune prophylaxis of rabies in domestic animals (7). This contributed to a high incidence of rabies in dogs (over 19% of all rabies cases) and in cats (over 25% of all cases) in UA (1).

In humans, a total of 36 rabies cases have been reported between 2007 and 2016 for UA, including the oblasts or cities of Donetsk, Kharkiv and Dnipro, Vinnytsia, Kyiv, Luhansk, Rivne, Ternopil, oblasts and Kyiv city, Volyn, Kirovograd, Lviv, Odesa, Poltava, Sumy, and Kherson (8).

A concerted effort has been set forth to curtail the spread of rabies in Europe. Oral rabies vaccination (ORV) of wild carnivores with live-attenuated or recombinant rabies vaccines proved to be a very effective measure in Europe (6, 9). Large-scale ORV campaigns started in 1985 considerably decreased rabies cases in Western Europe. The same was not true for Eastern Europe, where rabies is still in high prevalence, making disease management a critical issue in this part of the continent (1). ORV was introduced to UA under field conditions in the late 1990s. Due to limited financial resources, only areas with high rabies prevalence were targeted. In the spring of 2001, 80,000 doses of the rabies vaccine “RABIFOX®” (Impfstoffwerk Dessau Tornau GmbH, Rosslau, Germany) were distributed in six oblasts: Chernihiv, Sumy, and Luhansk oblasts (20,000 doses per oblast); Cherkassy (10,000 doses); and Kyiv and Poltava oblasts (5,000 doses each). Vaccines were distributed by foot mainly near foxholes. However, these measures did not decrease rabies cases (10). In 2001–2003, ORV was implemented in Odessa oblast. The vaccine contained the Vnukovo-32 strain (LLC Vidrodzhennya M, Odesa, Ukraine) (9) and vaccine-laced baits were distributed by foot mainly near foxholes. However, this effort was not successful in reducing rabies cases either. In fact, for 2002–2005, three vaccination campaigns were held in Poltava oblast using the Rabivak HTT (Sumy Biological Factory, Sumy, Ukraine) vaccine, and they were distributed by foot (11). The assessment after the campaign equally showed no decrease of rabies cases reported by State veterinary services in Poltava oblast. Subsequently (2003–2004), ORVs of foxes with the recombinant Raboral V-RG (Merial, Inc., Athens, Georgia, USA) vaccine was performed in Dzhankoi and Nyzhniohirsk rayons of the Autonomous Republic (AR) of Crimea. The study of this campaign showed a high vaccine consumption level among targeted animal types (over 95% for 15 days) and a high degree of immune protection among foxes, with corresponding decreases in rabies cases for these areas (12).

The successful international ORV campaigns using Raboral V-RG (Merial, Inc., Athens, Georgia, USA) for wild carnivores in Crimea (13, 14) instigated the beginning of large-scale ORV campaigns using Brovarabis V-RG (Ukrvetprompostach LLC, Brovary, Ukraine) recombinant vaccine in UA in December 2006. From 2006 to 2014, the ORV campaigns targeted 18 oblasts. However, the implementation of these campaigns was irregular with the exception of eastern Ukraine (i.e., in Kharkiv, Sumy, Poltava, Luhansk, and Donetsk oblasts) where the campaigns were implemented regularly. Upon conclusion of these campaigns, the number of rabies outbreaks decreased, especially in Poltava and Luhansk (6, 15).

ORV campaigns with Brovarabis V-RG (Ukrvetprompostach LLC, Brovary, Ukraine) were also implemented in the western oblasts of UA bordering Poland and Hungary (i.e., in Lviv, Volyn, and Zakarpattia) by 2012. The aim of these campaigns was to protect rabies-free territory in Poland and Hungary, given the transboundary nature of rabies, by creating a buffer area along the administrative border with UA under the framework of an interstate agreement (16, 17). Vaccine was distributed by air targeting those areas neighboring Poland and Hungary. A concentration of 25 baits/km^2^ was used. From 2012 to 2016, campaigns were implemented with progressively larger targeted areas (from about 26,000 km^2^ in 2012 to more than 48,000 km^2^ in 2016). Precise geo-informational systems (GIS) data on bait distribution were only available from 2016; from that point on, the automated system recorded the geographical coordinates of each bait. Moreover, an electronic metronome connected to a global positioning system (GPS Control Distribution Unit gen.4, GPS CDU-4) regulated the frequency of vaccine dropping accounting for airplane speed, providing a strong likelihood of homogeneous bait coverage (18).

In the present study, we hypothesize that the effectiveness of the ORV campaigns in western Ukraine is limited as shown by spatial analysis of rabies cases. Our first objective was to describe the spatial and temporal patterns of rabies in three Ukrainian oblasts (i.e., Lviv, Volyn, and Zakarpattia), where ORV campaigns in foxes were carried out in 2012–2016. Using the results of the analysis, we then attempted to discuss the effectiveness of the ORV in the targeted oblasts.

## Materials and Methods

### Study Area

**Figure 1:**
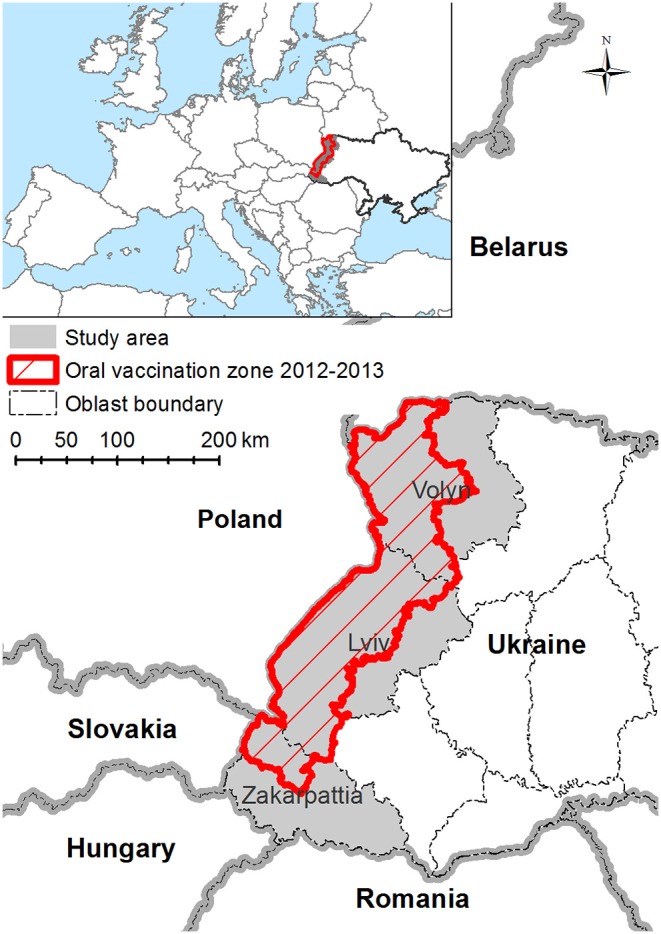
Map of study area—Volyn, Lviv, and Zakarpattia oblasts of Ukraine. Study area is marked with gray fill. Red shaded area is a territory where Ukraine performed the oral vaccination in years 2012–2013.

This study targets three western oblasts of UA, namely, Volyn, Lviv, and Zakarpattia. These oblasts border, from north to south, Belorussia, Poland, Slovakia, Hungary, and Romania (Figure 1) and comprise ~9% of the all Ukrainian territory. This region is scarcely populated, mostly mountainous, and covered by forests. The study area is described in Figure 1.

Volyn oblast is 20,200 km^2^ (3.3 % of the territory of UA) and characterized by two clearly distinguished types of landscapes: (a) the northern part with large forests, bog areas, and numerous lakes and (b) the southern part encompassing valleys and mountain chain reliefs with small forests. The area of forests of Volyn oblast is 6,980 km^2^. Lviv oblast is 21,800 km^2^ (3.6% of the territory of UA), out of which 6,950 km^2^ are forest areas. The Carpathian Mountains cover the southern part of the Lviv oblast. Zakarpattia oblast is 12,800 km^2^ (2.1% of the territory of UA), with about 80% being mountainous and 50% forests (6,570 km^2^).

### Disease Data

Rabies data on wild and domestic animals within the oblasts of Volyn, Lviv, and Zakarpattia were collected for the period between 2012 and 2016 by the state oblast veterinary laboratories in UA. Animal brain tissue samples were tested of rabies virus using Fluorescent Antibody Test (FAT) (19). During this investigation, the laboratory received a total of 10,560 animal samples, 427 of which were confirmed rabies cases (Table 1). The largest part (94.74%) of data on foxes was collected through active monitoring, i.e., hunter-killed foxes. Samples from other species were received through passive supervision of rabies, i.e., sent to oblast (Volyn, Lviv, and Zakarpattia) laboratories by officers of the state veterinary service (20).

**Table 1:** Rabies cases in Lviv, Volyn, and Zakarpattia oblasts 2012–2016.

**Species**	**Lviv**					**Volyn**					**Zakarpattia**					**Total**
	**2012**	**2013**	**2014**	**2015**	**2016**	**2012**	**2013**	**2014**	**2015**	**2016**	**2012**	**2013**	**2014**	**2015**	**2016**	
1) Foxes	27	18	10	17	14	22	40	21	23	24	4	2	1	3		226
2) Domestic carnivores	6	5	5	8	14	12	15	10	22	35	9	10	3	6	9	169
•Cat	2	2		4	9	8	9	6	14	22	3	4	3	3	2	91
•Dog	4	3	5	4	5	4	6	4	8	13	6	6		3	7	78
3) Other wild animals	1	3	1			4	3	3	5	1						21
•Bat							1									1
•Roe deer	1															1
•Marten		3				2		1	1							7
•Ferret			1				1		1							3
•Rat						1										1
•Raccoon dog						1	1	2	2							6
•Wolf									1	1						2
4) Other domestic animals		1						1	2	3	1	2			1	11
•Cow		1							1	2	1	1			1	7
•Goat									1							1
•Guinea pig								1								1
•Horse										1		1				2
Total per oblast	130					246					51					427

*Total number of cases/species category is shown in gray shaded areas*.

The data collected into our database system included animal species, coordinates of the centroid of the geographical area from which the sample was received, and date of sample acceptance by the testing laboratory (pathologic material).

If GPS data were not available, the centroid of the nearest village was calculated using sample origin data.

### Descriptive Statistical Analyses

Rabies cases were classified into four groups: (1) foxes, (2) domestic carnivores (i.e., cats and dogs), (3) other wild animals (including roe deer, marten, ferret, rat, raccoon dog, bat, and wolf), and (4) other domestic animals (i.e., cow, goat, horse, and guinea pig) (Table 1). Data on foxes were separated from other species as foxes are believed to be a major reservoir of rabies in Europe (3, 4). Similarly, dogs and cats were grouped together, as they are considered the primary source of rabies virus in human infections in UA (8). Statistical analysis, therefore, focused on these two categories. However, descriptive results for the other groups were also reported in Table 1.

The percentage of positive samples was calculated as the number of positive samples divided by the total number of samples analyzed in the group from the entire study area (Table 2) or from each of the oblasts (Table 3). The exact binomial confidence intervals (BCIs) for the percentage of positive values were calculated using Epitools package in R (21).

**Table 2:** Rabies cases by year (2012–2016) with 95% exact binomial confidence interval.

**Species**	**Year**	**Positive**	**Samples**	**Percentage of positive**	**Lower 95% BCI**	**Upper 95% BCI**
Foxes	2012	53	1,220	4.34	3.27	5.64
	2013	60	2,493	2.41	1.84	3.09
	2014	32	2,024	1.58	1.08	2.22
	2015	43	1,661	2.59	1.88	3.47
	2016	38	1,418	2.68	1.90	3.66
Domestic carnivores (cats and dogs)	2012	27	287	9.41	6.29	13.39
	2013	30	267	11.24	7.71	15.65
	2014	18	226	7.96	4.79	12.30
	2015	36	289	12.46	8.88	16.83
	2016	58	385	15.06	11.64	19.04
Other domestic animals	2012	1	12	8.33	0.21	38.48
	2013	3	8	37.50	8.52	75.51
	2014	1	7	14.29	0.36	57.87
	2015	2	12	16.67	2.09	48.41
	2016	4	18	22.22	6.41	47.64
Other wild animals	2012	5	43	11.63	3.89	25.08
	2013	6	65	9.23	3.46	19.02
	2014	4	39	10.26	2.87	24.22
	2015	5	43	11.63	3.89	25.08
	2016	1	43	2.33	0.06	12.29

**Table 3:** Rabies cases by year (2012–2016) for each oblast with 95% exact binomial confidence interval.

**Species**	**Year**	**Positive**	**Samples**	**Percentage of positive**	**Lower 95% BCI**	**Upper 95% BCI**
**Volyn oblast**						
Foxes	2012	22	280	7.86	4.99	11.65
	2013	40	892	4.48	3.22	6.06
	2014	21	685	3.07	1.91	4.65
	2015	23	444	5.18	3.31	7.67
	2016	24	464	5.17	3.34	7.60
Domestic carnivores (cats and dogs)	2012	12	101	11.88	6.29	19.83
	2013	15	92	16.30	9.42	25.46
	2014	10	65	15.38	7.63	26.48
	2015	22	94	23.40	15.29	33.26
	2016	35	180	19.44	13.93	25.99
Other domestic animals	2012	0	3	0.00	0.00	70.76
	2013	0	2	0.00	0.00	84.19
	2014	1	4	25.00	0.63	80.59
	2015	2	6	33.33	4.33	77.72
	2016	3	11	27.27	6.02	60.97
Other wild animals	2012	4	11	36.36	10.93	69.21
	2013	3	15	20.00	4.33	48.09
	2014	3	9	33.33	7.49	70.07
	2015	5	17	29.41	10.31	55.96
	2016	1	16	6,25	0.16	30.23
**Lviv oblast**						
Foxes	2012	27	840	3.21	2.13	4.64
	2013	18	1,426	1.26	0.75	1.99
	2014	10	1,167	0.86	0.41	1.57
	2015	17	1,090	1.56	0.91	2.49
	2016	14	842	1.66	0.91	2.77
Domestic carnivores (cats and dogs)	2012	6	153	3.92	1.45	8.34
	2013	5	138	3.62	1.19	8.25
	2014	5	139	3.60	1.18	8.19
	2015	8	167	4.79	2.09	9.22
	2016	14	183	7.65	4.25	12.50
Other domestic animals	2012	0	8	0.00	0.00	36.94
	2013	1	3	33.33	0.84	90.57
	2014	0	3	0.00	0.00	70.76
	2015	0	6	0.00	0.00	45.93
	2016	0	4	0.00	0.00	60.24
Other wild animals	2012	1	25	4.00	0.10	20.35
	2013	3	44	6.82	1.43	18.66
	2014	1	29	3.45	0.09	17.76
	2015	0	24	0.00	0.00	14.25
	2016	0	22	0.00	0.00	15.44
**Zakarpattia oblast**						
Foxes	2012	4	100	4.00	1.10	9.93
	2013	2	175	1.14	0.14	4.07
	2014	1	172	0.58	0.01	3.20
	2015	3	127	2.36	0.49	6.75
	2016	0	112	0.00	0.00	3.24
Domestic carnivores (cats and dogs)	2012	9	33	27.27	13.30	45.52
	2013	10	37	27.03	13.79	44.12
	2014	3	22	13.64	2.91	34.91
	2015	6	28	21.43	8.30	40.95
	2016	9	22	40.91	20.71	63.65
Other domestic animals	2012	1	1	100.00	2.5	100.00
	2013	2	3	66.67	9.43	99.16
	2014	0	0	–	–	–
	2015	0	0	–	–	–
	2016	1	3	33.33	0.84	90.57
Other wild animals	2012	0	7	0.00	0.00	40.96
	2013	0	6	0.00	0.00	45.93
	2014	0	1	0.00	0.00	97.50
	2015	0	2	0.00	0.00	84.19
	2016	0	5	0.00	0.00	52.18

The percentage difference of positives in each group in the entire study area across the years was assessed using Pearson's chi-squared test in R (χ^2^). A statistically significant threshold was set at a *p* value of 0.05. This test was not performed in groups 3 and 4 (other wild animals and other domestic animals) due to the limited number of rabies-positive cases.

The Wilcoxon rank sum test in R was used to compare percentage of positive in the groups across the years among oblasts. A statistically significant threshold was set at a *p* value of 0.05. The test was performed only for the fox and the domestic carnivores groups.

**Figure 2:**
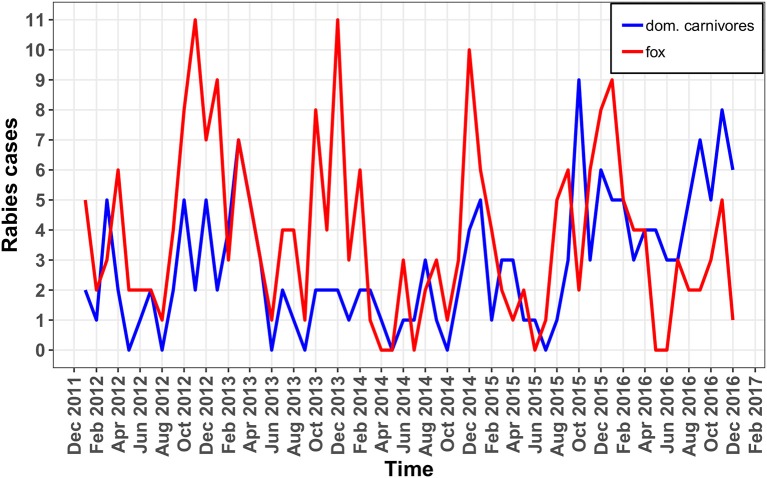
Epidemic curves of rabies in foxes (red line) and domestic carnivores (blue line) in monthly period.

Epidemic curves of rabies cases among foxes, cats, and dogs have been plotted showing the monthly incidence for all oblasts (Figure 2).

### Spatial and Temporal Analysis

To assess the dynamics and dispersion of rabies cases in the Ukrainian territory under investigation, we calculated the degree to which feature (i.e., positive cases) is concentrated or dispersed around the geometric mean center. The analysis was performed using the Standard Distance tool implemented in ArcGIS 10.3 (22). This method draws circles equal to standard deviations of the events distribution around their geometrical average center. We compared changes in the degree of dissemination of rabies cases among foxes, cats, and dogs on an annual basis, and we assessed changes across the years in the average geometrical center of rabies cases in those groups. The circle size of the standard distance was the default value of 1 standard deviation.

To investigate changes in the density of rabies cases among foxes, cats, and dogs in the oblasts under investigation on an annual basis, we estimated the Kernel density (KDE) from the set of Spatial Analyst tools in ArcGis 10.3. KDE provides a spatially smooth estimate of the local intensity of events (23). We calculated the search radius for smoothing according to Fotheringham et al. (24):

$$\begin{array}{l} h_{opt}={\left[\frac{2}{3n}\right]}^{1/4}\sigma \end{array}$$

where *n* = number of rabies cases and σ = standard distance of locations with rabies cases.

To compare densities of disease cases on an annual basis, we used the averaged *h_opt_* for all the years. A 1,000 m/pixel spatial resolution was chosen for all output rasters. It is the round-off of the default value suggested for our data by the KDE tool in ArcGIS. The spatial resolution influences the visual attractiveness of KDE output raster with a higher resolution (25).

We estimated the geographical territories where the kernel density estimation of rabies cases among foxes, cats, and dogs remained >0 during all years of observation. For this purpose, map algebra expression with boolean AND operator from the set of tools Spatial Analyst ESRI ArcGis 10.3 was applied to KDE rasters in the raster calculator.

To detect spatial–temporal clusters of rabies cases, we used a space–time permutation model implemented in SatScan (26). This model does not require information about the distribution of the underlying population, only data on disease cases (27). Because the model assumes that the spatial distribution of the population holds steady during the whole investigation period, we conducted the analysis separately for each year. For the analysis, the following parameters were chosen: maximum spatial size of the cluster−50% of the population at risk; maximum temporal size of the cluster−50% of the investigation period; time precision—day; scan for areas with—high rates; time aggregation-−1 day; *p* values for detected clusters—default, 999 Monte Carlo replications; no geographical overlap. To identify clusters of cases among foxes and domestic carnivores, we implemented two approaches: (1) applied consolidated data of all the groups in the analysis and (2) analyzed foxes and domestic carnivores groups separately.

All maps were generated in ESRI ArcGis 10.3 using projection UTM WGS 1984 zone 35N. Vector layers of countries' borders and administrative units GADM were used (http://gadm.org/).

## Results

### Descriptive Statistical Analysis

A total of 427 rabies cases were confirmed among domestic and wild animals from 2012 to 2016. Of those 427 cases, 226 (52.93%) were detected in foxes. Rabies in other wild animals were detected in 21 cases (4.92%). Among domestic carnivores (cats and dogs), 169 rabies cases (39.58%) were confirmed. This group represents the second largest group affected by the disease in the targeted oblasts. Eleven cases (2.57%) were detected in other domestic animals (Table 1).

The epidemic curve (Figure 2) in **foxes** shows a consistent increase in incidence in October through December of each year. The percentage of positive cases in foxes differed across the years of observation (χ^2^ = 23.637, df = 4, *p* < 0.05). The lowest percentage of positive rabies cases in foxes was observed in 2014 and the highest percentage was observed in 2012. From 2012 to 2014, there was a reduction in the percentage of positive rabies cases in foxes (2012−4.34%, 2013−2.41%, 2014−1.58%). That was followed by an increase the following 2 years (2015−2.59%, 2016−2.68%) (Table 2). The percentage of positive rabies cases in foxes across the years in Volyn oblast is significantly higher than that in Lviv and Zakarpattia oblasts based on a Wilcoxon rank sum test (*w* = 24, *p* < 0.01), while the difference is not statistically significant between Lviv and Zakarpattia oblasts.

The epidemic curve for domestic cats and dogs shown in Figure 2 displays a less regular pattern in comparison to foxes with no evident differences across the years of observation (χ^2^ = 8.9906, df = 4, *p* = 0.06134). The lowest percentage of positive rabies cases was observed in 2014 (similar to foxes), and the highest percentage of positive cases was observed in 2016 (Table 2). The percentage of positive cases in Volyn and Zakarpattia oblasts are significantly higher than in the Lviv oblast (*w* = 25, *p* < 0.01), while the percent difference is not statistically significant between Volyn and Zakarpattia oblasts.

### Spatial and Temporal Analysis

**Figure 3:**
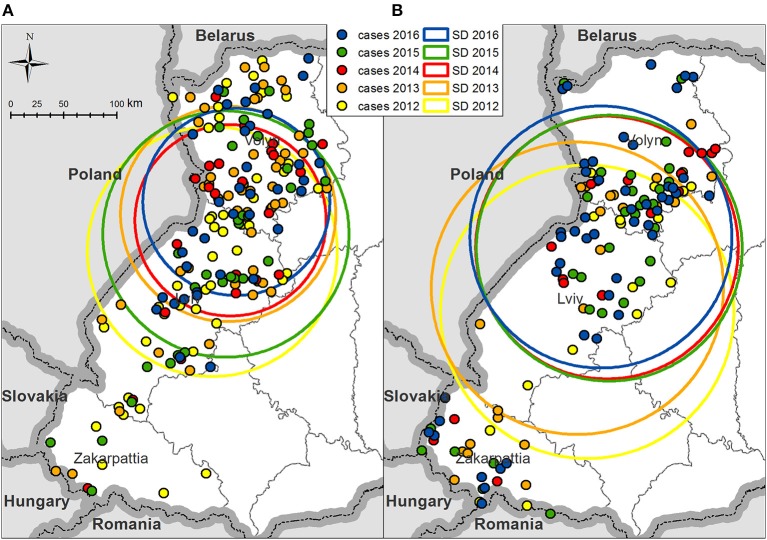
Standard distances of rabies cases in Volyn, Lviv, and Zakarpattia oblasts on an annual basis **(A)** in foxes and **(B)** in domestic carnivores. Colored circles represent 1 standard deviation of rabies cases dispersion in foxes or domestic carnivores for the corresponding year. The center of each circle is the averaged geographical coordinates of all cases of rabies in foxes or domestic carnivores for each year.

From 2012 to 2016, the average geographical center of rabies cases in foxes appears to have been shifted toward the north by 52 km; however, the standard distance, which represents the degree of their dispersion around the average center, decreased from 117.7 km in 2012 to 90.1 km in 2014 (Figure 3 and Supplemental Figure 1). The consistent reduction in dispersion (116 km) and shifting to the north (21 km) was not observed in 2015, because rabies cases in foxes in Zakarpattia and Lviv oblasts increased. In 2016, most rabies cases in foxes concentrated in the south and in the center of Volyn and north of Lviv oblasts.

The spatial pattern for domestic dogs and cats resembles that for the foxes, with a progressive shifting of the average center of rabies cases to the north and a reduction in the degree of their dispersion from 2012 to 2016. Rabies cases in dogs are less concentrated around the average center but are more widely distributed over the territories of oblasts (Figure 3 and Supplemental Figure 2).

**Figure 4:**
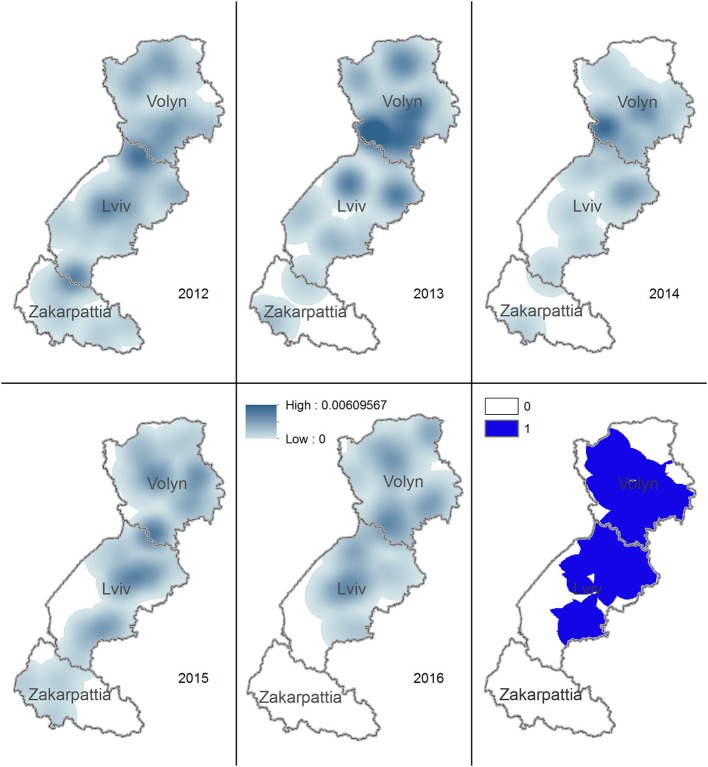
Kernel density estimation of rabies cases in foxes in Volyn, Lviv, and Zakarpattia oblasts. Yearly maps representing the areas with higher density of rabies cases in foxes for years 2012–2016 are shown. Solid blue color on the bottom right figure marks the territory where the density of cases is consistently >0 across all years.

The density of rabies cases in foxes was noticeably reduced in Lviv and Zakarpattia oblasts in 2014. In 2016, the density was zero for the whole territory of Zakarpattia oblast. The territory having density of rabies cases in foxes during 2012–2016 > 0 covers most of the Volyn and Lviv oblasts (Figure 4).

**Figure 5:**
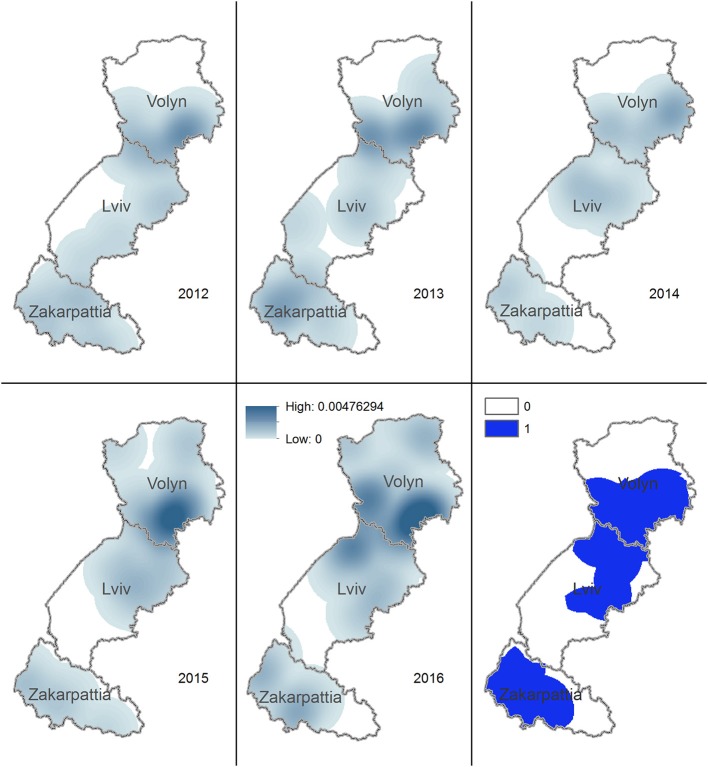
Kernel density estimation of rabies cases in domestic carnivores in Volyn, Lviv, and Zakarpattia oblasts. Yearly maps representing the areas with higher density of rabies cases in domestic dogs and cats for years 2012–2016 are shown. Solid blue color on the bottom right figure marks the territory, where the density of cases is consistently >0 across all years.

The highest density of rabies cases in dogs and cats during 2012–2016 is on the border of Volyn and Lviv oblasts. The territory where the density of rabies cases in dogs and cats used to be >0 during 2012–2016 spreads from the center of Volyn to the center of Lviv oblast and covers most of the area of Zakarpattia oblast (Figure 5).

**Figure 6:**
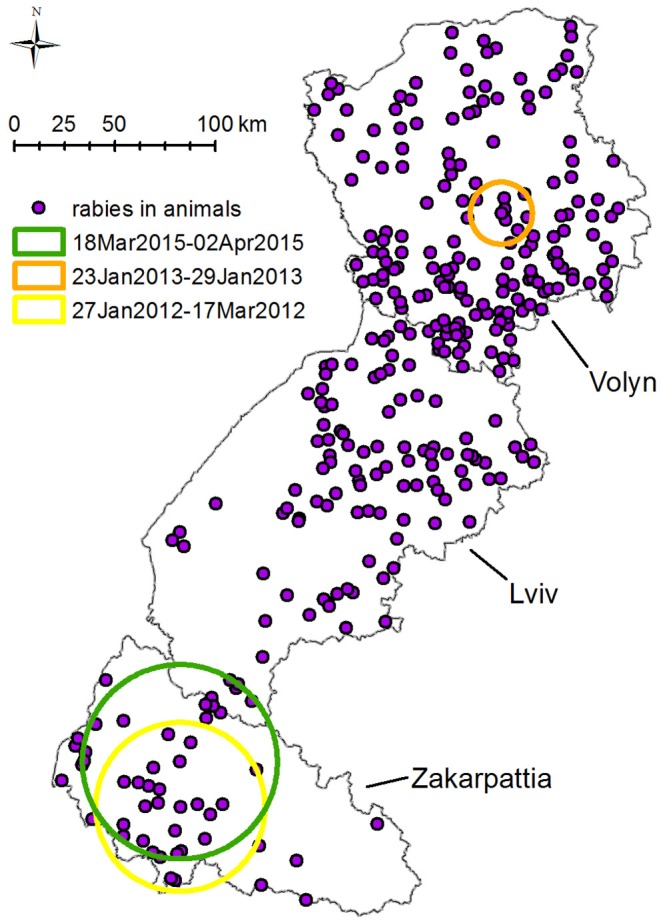
Spatiotemporal clustering of rabies cases in all species in Volyn, Lviv, and Zakarpattia oblasts from 2012 to 2016 detected using space–time permutation model in SatScan. Three spatiotemporal clusters with *p* < 0.05 were detected: (1) yellow circle: 41.9 km radius—present in 2012 from January 27 to March 17, displaying seven cases of rabies (two in foxes, three in dogs, and two in cats). (2) Orange circle: 15.4 km radius—present in 2013 from January 23 to January 29, displaying five cases of rabies four in foxes and one in raccoon dog. (3) Green circle: 48.2 km radius—present in 2015 from March 18 to April 2, displaying five cases of rabies (three in foxes and two in cats).

SatScan space–time permutation model performed for all species has found three statistically significant clusters with *p* < 0.05 of rabies cases. The earliest cluster (*p* = 0.01, from January 27, 2012, to March 17, 2012) is located south of Zakarpattia oblast at the border with Hungary and Romania with a radius of 41.9 km (Figure 6). This cluster includes seven cases of rabies in animals: two in foxes, three in dogs, and two in cats.

The second cluster (*p* = 0.022, January 23 to January 29, 2013) with a radius of 15.4 km is situated in the center of Volyn oblast, at the boundary of the oral vaccination zone of 2012–2013 (Figure 6). This cluster includes five cases of rabies (four in foxes, one in raccoon dog). The third cluster with *p* = 0.028 is located in Zakarpattia oblast; its center is situated to the north of the first cluster center. The radius of the cluster is 48.2 km and spans from March 18, 2015, until April 02, 2015. It includes five cases of rabies (three in foxes, two in cats).

**Figure 7:**
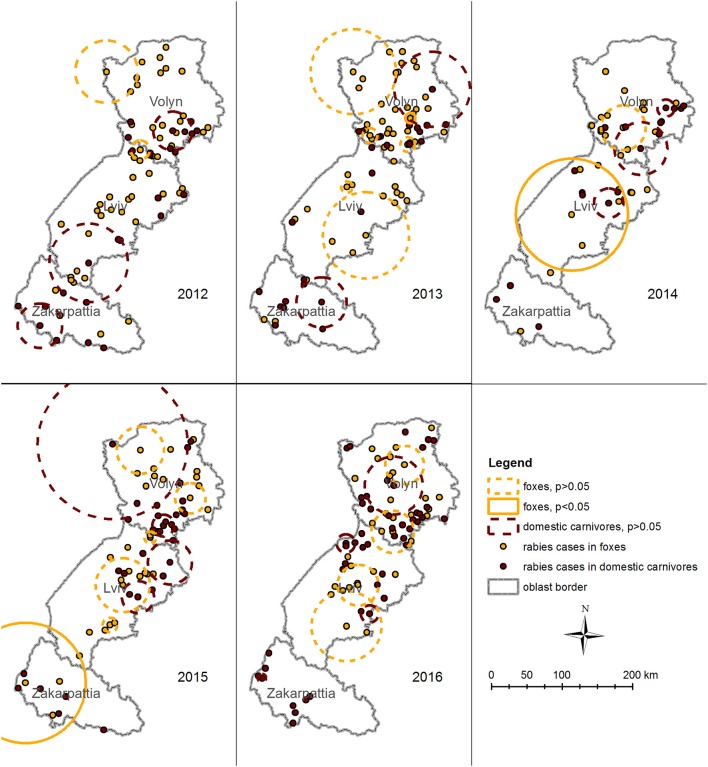
Spatiotemporal clustering of rabies cases in foxes (orange circles) and domestic carnivores (brown circles) in Volyn, Lviv, and Zakarpattia oblasts from 2012 to 2016 detected using space–time permutation model in SatScan. Solid circles represent clusters with *p* < 0.05. Dashed circles represent clusters with *p* > 0.05. Space–time permutation model for rabies cases in domestic carnivores did not detect a significant cluster. Significant clusters of rabies cases in foxes were detected for years 2013, 2014, and 2015. Although some of them overlap with insignificant clusters in domestic carnivores in space, none of them intersect in time.

Space–time permutation model performed for rabies cases in foxes detected three likely clusters with *p* < 0.05 in 2013, 2014, and 2015. The earliest cluster (*p* = 0.04, January 24, 2013) is located close to the center of Volyn oblast and has a radius of 7.2 km. It comprises of three cases (Figure 7) and intersects in space and time for all species. The second cluster (*p* = 0.016, February 17 to June 19, 2014) is located in Lviv oblast with a radius of 79.3 km and comprises five cases. The third cluster (*p* = 0.005, March 18 to May 12, 2015) is located in the western part of Zakarpattia oblast with a radius of 85.3 km. It consists of four cases and intersects in space and time for all species.

Space–time permutation model for rabies cases in domestic carnivores did not detect a significant cluster (Figure 7). However, across the years (from 2012 to 2016) a number of clusters of different size were identified among other species. From 2012 to 2013, relatively large clusters were identified in Zakarpattia oblast, in the southern part of Lviv oblast, and in Volyn oblast. In the years following (2014–2016), clusters were not identified in Zakarpattia oblast, when most clusters were detected in northern Lviv and in Volyn oblasts.

Although, some of the clusters in foxes and domestic carnivores overlap in space, none of them intersect in time.

## Discussion

The surveillance efforts implemented in Volyn, Lviv, and Zakarpattia oblasts from 2012 to 2016 show a permanent detection of rabies in the targeted oblasts in both wild animals (predominantly foxes) and domestic animals (predominantly dogs and cats). The percentage of positive rabies cases is different across oblasts, with Zakarpattia apparently less affected. This may be caused by the environmental features of the oblast with more than 80% of the territory covered by mountains, which might have resulted in lower reporting frequency.

As expected, a large proportion of rabies cases were found in foxes (i.e., 52.93%). The role of foxes in the epidemiology of rabies in UA and other European countries is well-documented (3, 4, 13). Consistent increase in incidence of rabies in foxes in October through December of each year is likely connected with the ecology of foxes, as young foxes tend to leave the foxhole in autumn to expand into new territories (28). This may result in increased contacts with other foxes and livestock contributing to the spread of rabies. The high incidence reported in foxes in this study is consistent with other research (10–12, 15). The surveillance strategy in UA relies heavily on the reporting and sampling of hunted foxes rather than other strategies likely to be more effective (29). This caused a higher number of fox samples being submitted to the laboratories and lower percentage of positive samples compared to other species (see Table 2). This is likely because, as anticipated in the methods section, species other than foxes are sampled based on the suspicion of the disease enhancing the specificity of the approach. For this reason, the rabies percentage of positive among foxes more likely represents the actual rabies-infected fox population within the area under investigation.

Rabies cases in both foxes and domestic carnivores appeared to shift toward northern territories from 2012 to 2016. This pattern may be explained by the reduction in the number of rabies cases in foxes in the southernmost oblasts, i.e., Zakarpattia oblast where no cases in foxes were recorded in 2016. Geometrical centers of rabies cases for foxes, dogs, and cats in 2012 through 2016 are located on the border between Volyn and Lviv oblasts. This may be explained by the highest concentration of the human population and consequently by a higher population of domestic animals (dogs and cats). In addition, the synantropization of foxes (i.e., approaching of habitats to the populated areas), a well-documented pattern (30), might have led to an increase in the fox population in this area, enhancing the chance for transmission. The evaluation of the density of rabies cases in foxes demonstrated a reduction across 2014–2016 in the southern part of Lviv oblast and in Zakarpattia oblast. However, the density of rabies cases in foxes on a north part of Lviv and Volyn oblasts has remained always >0 in 2012–2016.

The epidemiological and spatial analysis confirmed the presence of areas with a high density of cases and spatial and temporal clusters of disease in different species across 2012–2016 and in territories covered with the ORV in Volyn, Lviv, and Zakarpattia oblasts. This implies a wide circulation and transmission of the virus between species in the areas and casts strong doubts on the efficacy of the vaccination campaigns implemented in western Ukraine. Effective examples of ORV campaigns are well-documented (6, 9, 14, 29, 31, 32). In many European countries, ORV had reduced rabies cases by 90% in 5–10 years. Variation in the reduction of rabies cases detected after each ORV is dependent on multiple factors such as geographical location, initial epidemiological situation, tools and strategy used within the control programs, and implementation of an appropriate surveillance plan (33). In UA, ORV in foxes performed for 5 years (2012–2016) in Volyn, Lviv, and Zakarpattia oblasts seems to have had little influence on the endemic manifestation of rabies in the territories. This could have been caused by several factors: improper and irregular implementation ORV campaigns (with only one campaign launched in autumn of 2015 and another one in 2016), weather-related delays in vaccine delivery, considerable distances between airplane flight lines (particularly over mountainous territories), and use of vaccine airdrop strategies (the use of an electronic metronome with GPS started only in 2016). These factors and others might have influenced an uneven and poorly effective distribution of the vaccine baits in the territory. In contrast, campaigns implemented in eastern Ukraine (Kharkiv, Sumy, Poltava, Luhansk, and Donetsk oblasts), which were conducted regularly, resulted in a noticeable decrease in the incidence of rabies cases, especially in the Poltava and Luhansk oblasts (6, 15). The State veterinary service in Ukraine is attempting to improve the efficacy of the vaccination campaigns. To improve control of distribution of vaccine, all the airplanes that implemented the campaign in 2017–2019 were equipped with an electronic metronome connected to a GPS.

Detection of three disease clusters including cases in both wild and domestic animals (Figure 6) [although not supported by epidemiological or molecular (sequencing) evidence] and the presence of clusters in foxes and domestic carnivores close to one another (Figure 7) suggest possible cross-species transmission from foxes to dogs and cats. In addition, our findings confirm that statistically significant clusters of foxes preceded in time the clusters of all other species. However, more robust observational and genetic studies should be implemented to prove the interspecies transmission (34).

The percentage of positive rabies cases among dogs and cats (39.58%) indicates an insufficient level of immunity against rabies in these populations. This may be associated with an inappropriate adoption of the national legislation that regulates the preventive vaccination of domestic animals by dog owners. According to the Ukrainian instruction “Preventive measures against rabies of animals” (20), all dogs must be vaccinated against rabies. Nychyk (7) have shown that, out of 228 cases of rabies in dogs, only 26 of those dogs (12.9%) were stray, while the majority (202; 87.1%) had owners, but didn't get the rabies vaccination prescribed by the national regulation. In addition, serological analysis of 234 dog samples indicated a population immunity of 36.6% in Ukrainian towns, and 9.1% in villages (7). The reasons for this are poor rabies awareness in remote areas, or poor availability of vaccines in remote veterinary facilities. Further studies could confirm this hypothesis and authorities should guarantee the vaccines' availability, especially in high-risk areas (areas with increased incidence or with clusters of disease).

## Conclusion

The study of rabies endemic outbreaks in western Ukraine (Volyn, Lviv, and Zakarpattia oblasts) from 2012 to 2016, in an area where an ORV program targeted to foxes existed, has demonstrated insufficient campaign efficacy.

The inclusion of dogs and cats into the endemic outbreak has demonstrated a deficiency of the current rabies prevention system among domestic animals. This indicates the need to identify and mitigate shortcomings to provide maximum rabies vaccine coverage of dogs and cats.

The rabies outbreak surveillance system in UA currently does not collect GIS information, which is critical to identifying disease distribution and potential disease cluster presence in relation to ecologic and land use features.

The creation of a national database of rabies cases in UA, which also stores GIS information, is currently under development. The first contribution to this database has been previously discussed by Polupan et al. (35), presenting the results of the first investigation of rabies endemic outbreak with the help of GIS in Chernihiv oblast. Our study contributed to this investigation by gathering all data (with GIS information) related to rabies cases in Volyn, Lviv, and Zakarpattia oblasts. We believe that the national database for rabies will become an efficient information resource in the future. Determining the spatial trends and identification of rabies clusters can be useful for making efficient distribution of effort decisions to control the disease.

## Data Availability

The datasets for this manuscript are not publicly available because all data generated or analyzed during this study are included in this published article and its Supplementary Information files. Requests to access the datasets should be directed to Ivan Polupan, i.n.polupan@gmail.com.

## Author Contributions

IP and MB contributed to the concept, design, data analysis, and interpretation, and drafted the current version of the paper. MD contributed to the concept, design, data interpretation, and the preparation of the manuscript. YG, ZD, and OR contributed to collection and primary analysis of data. VU oversaw data design and helped in data analysis and manuscript proofreading. VN contributed to the data analysis and interpretation. All authors contributed to the writing of, reviewed, and approved the final manuscript.

### Conflict of Interest Statement

MD is employed by SAFOSO, a Swiss SME involved in veterinary and food safety research projects. The remaining authors declare that the research was conducted in the absence of any commercial or financial relationships that could be construed as a potential conflict of interest.

## Supplementary Material

Supplemental Figure 1 Spatial distribution of rabies cases in foxes by year. Mean center (black cross) of rabies cases shows spatial shift of the distribution across the years 2012–2016. Standard distance circles represent changes in dispersion of rabies cases in foxes across the years.

Supplemental Figure 2 Spatial distribution of rabies cases in domestic carnivores by year. Mean center (black cross) of rabies cases shows spatial shift of the distribution across years 2012–2016. Standard distance circles represent changes in dispersion of rabies cases in domestic carnivores across the years.
