# Supplementary figures and images for: An Analysis of Rabies Incidence and Its Geographic Spread in the Buffer Area Among Orally Vaccinated Wildlife in Ukraine From 2012 to 2016
Source: Front Vet Sci. 2019 Sep 10;6:290. doi: 10.3389/fvets.2019.00290 (PMC6748165; doi:10.3389/fvets.2019.00290)

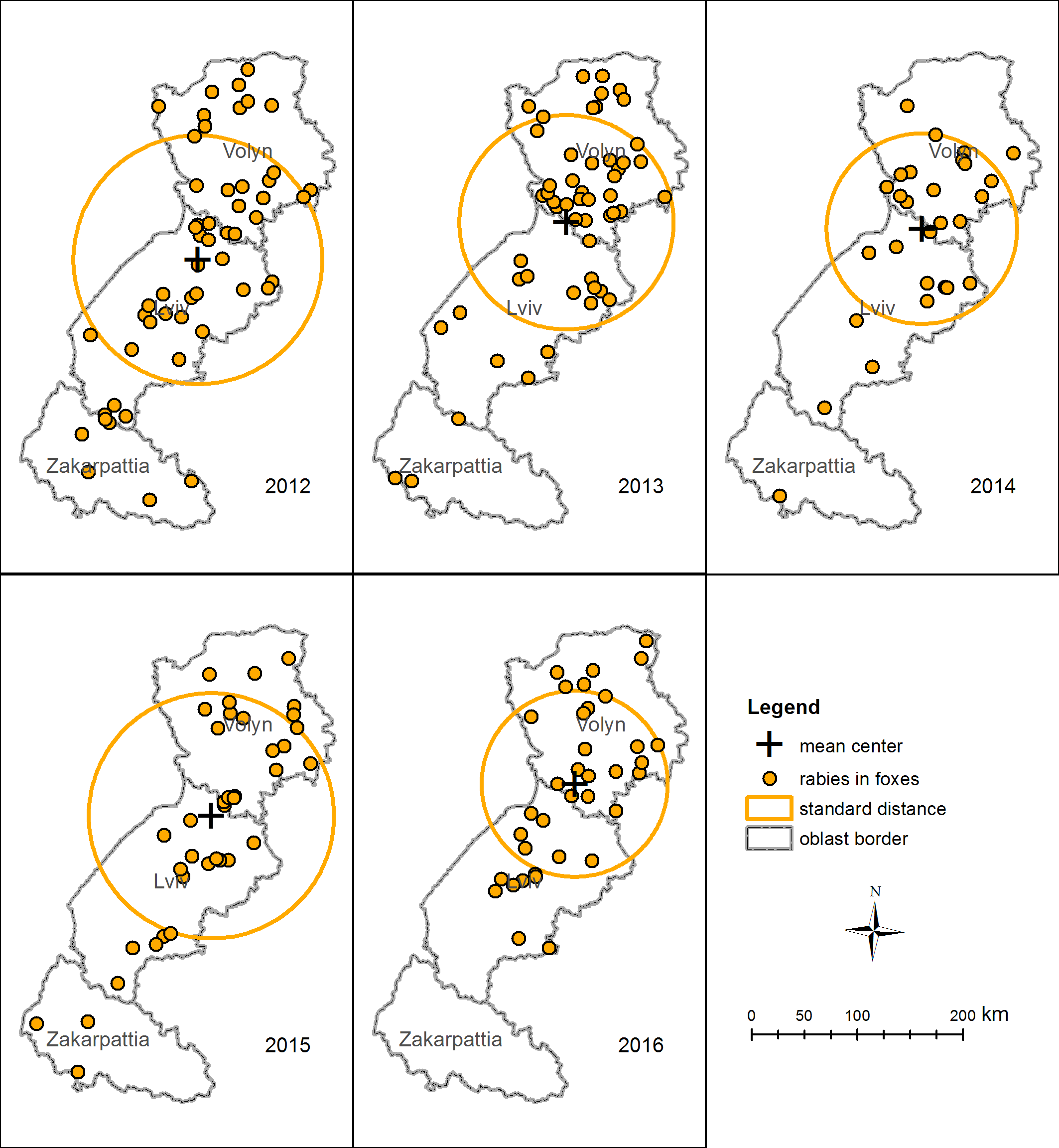

Supplement: Supplemental Figure 1 — Spatial distribution of rabies cases in foxes by year. Mean center (black cross) of rabies cases shows spatial shift of the distribution across the years 2012–2016. Standard distance circles represent changes in dispersion of rabies cases in foxes across the years. [file Image_1.TIF]

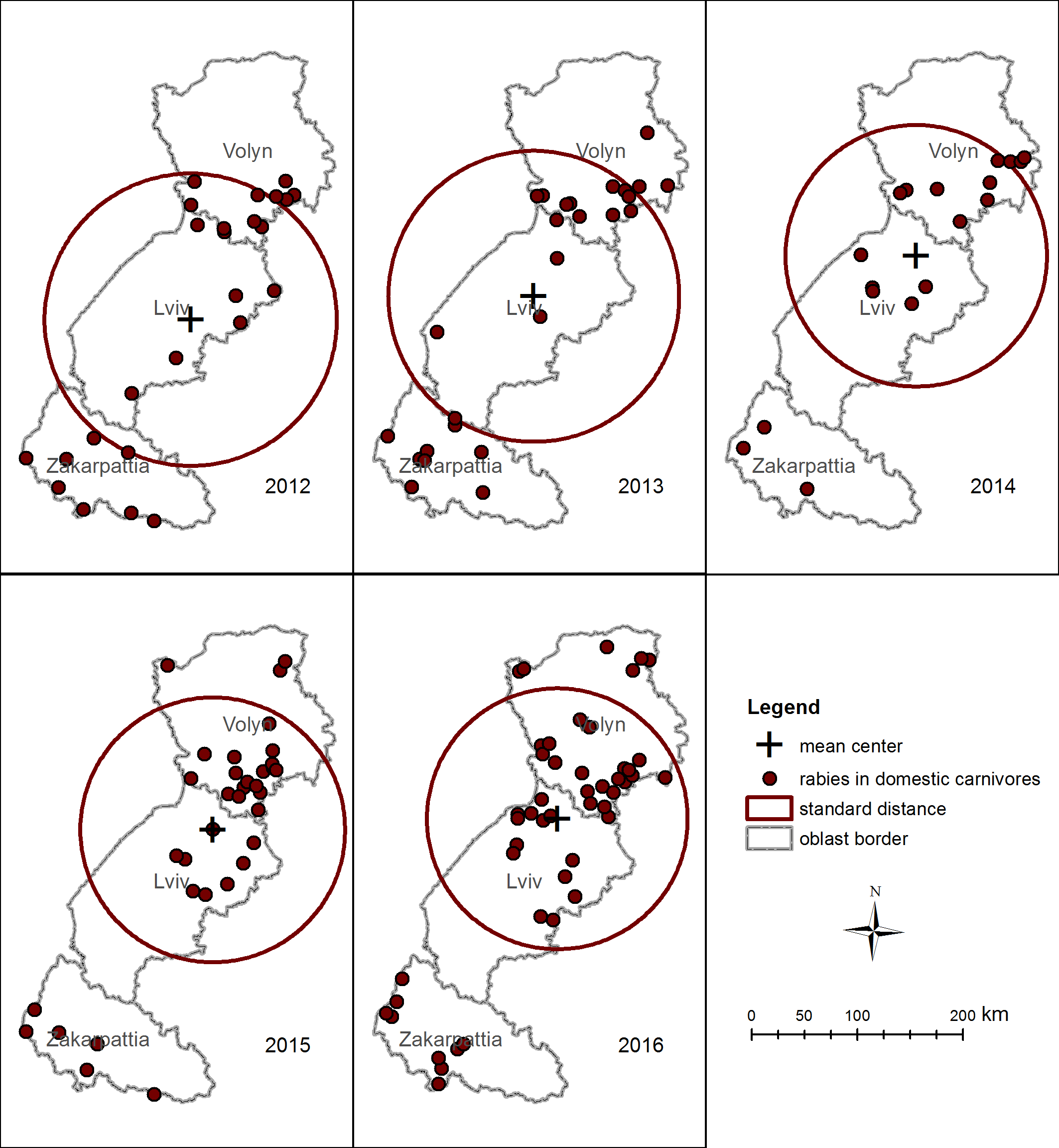

Supplement: Supplemental Figure 2 — Spatial distribution of rabies cases in domestic carnivores by year. Mean center (black cross) of rabies cases shows spatial shift of the distribution across years 2012–2016. Standard distance circles represent changes in dispersion of rabies cases in domestic carnivores across the years. [file Image_2.TIF]
